# Fruits and vegetables dietary intake and its estimated consumption among adults receiving antiretroviral therapy in health facilities in Northcentral Ethiopia: a multi-facility cross-sectional study

**DOI:** 10.3389/fnut.2024.1380987

**Published:** 2024-07-17

**Authors:** Dube Jara Boneya, Ahmed Ali Ahmed, Alemayehu Worku Yalew, Samson Gebremedhin

**Affiliations:** School of Public Health, College of Health Sciences, Addis Ababa University, Addis Ababa, Ethiopia

**Keywords:** fruit, vegetable, HIV/AIDS, dietary intake, antiretroviral therapy, adults

## Abstract

**Background:**

Despite the significant role of fruit and vegetables (FAVs) in preventing a variety of chronic diseases and their potential to bolster immune responses and slow the progression of HIV infection to AIDS, there is a lack of studies on the dietary intake of FAVs among HIV-infected adults in Africa, including Ethiopia. Hence, this study aimed to investigate the magnitude of FAV intake and estimated consumption among HIV-infected adults receiving antiretroviral therapy (ART) in northcentral Ethiopia.

**Methods:**

A multifacility cross-sectional study was conducted on the FAV intake among 865 HIV-infected adults receiving ART. A Poisson regression model with robust variance was used to identify factors associated with FAVs dietary intake.

**Results:**

The study indicated that 655 (76.34%; 95% CI: 73.38, 79.07) HIV-infected adults reported consuming FAVs less than once per day, with 838 (97.67%, 95% CI: 96.41, 98.49) and 676 (78.79%, 95% CI: 75.92, 81.40) HIV-infected adults reporting consuming fruits and vegetables less than once per day, respectively. The median (IQR) total FAV intake was 271.3 (IQR: 92.5, 439.5) g/day, with the median (IQR) intake of fruits being 248.1 (IQR: 100.0, 400.0) g/day and vegetables being 273.78 (IQR: 82.44, 348.33) g/day, respectively. We found that being divorced (APR = 1.57, 95% CI: 1.16, 2.12), employed as a daily laborer (APR = 2.08, 95% CI: 1.36, 3.20), being employed (APR = 1.77, 95% CI: 1.10, 2.84), merchants (APR = 1.59, 95% CI: 1.03, 2.47), having children as caregivers (APR = 1.61, 95% CI: 1.02, 2.55), an advanced WHO clinical stage (APR = 1.32, 95% CI: 1.32(1.03, 1.69), and receiving ART for more than 8 years (APR = 1.78, 95% CI: 1.18, 2.67) were found to be independent predictors of FAV dietary intake among HIV-infected adults. From the findings, we understood that farmers were less likely to consume FAVs compared to employed individuals, daily laborers, and merchants.

**Conclusion:**

The finding indicated a very low level of FAV dietary intake among HIV-infected adults receiving ART, falling well-below the minimum recommendation for physically active adults. Despite living in areas with surplus production and producing these items, farmers are less likely to consume FAV. The study emphasizes the importance of focusing on the early stage of ART treatment for patients and family therapy, including counseling and guidance on consuming healthy diets such as FAVs, to enhance the role of children as caregivers for their families. Additionally, there is a need for comprehensive nutritional counseling to improve FAV consumption, with a particular emphasis on educating individuals about portion size estimation for the consumption of FAVs.

## Introduction

In 2022, approximately 1.3 million new HIV infections were recorded globally, exceeding the global targets by over one million (1–3). Despite the availability of effective HIV treatments and tools for preventing, detecting, and treating opportunistic infections, the AIDS pandemic claimed a life every minute, resulting in 500,000 AIDS-related deaths in 2022 (2–5).

Good nutrition should be integrated into the care and treatment plan to enhance treatment success and improve the quality of life among HIV-infected individuals. A well-balanced diet can contribute to achieving a healthy weight gain, strengthening the immune system, preventing infection, and reducing hospital stays. It also helps the body build and maintain muscle mass, enhances the effectiveness of medications, aids in managing the side effects of medication, and improves the overall quality of life (6, 7). Fruits and vegetables (FAVs) are important components of a healthy diet and good nutrition. The consumption of FAVs could help prevent a variety of chronic communicable and non-communicable diseases, including HIV/AIDS (8).

HIV-infected patients have compromised immune systems, leading to inflammation and an increased risk of chronic diseases and infections. They require balanced portions of fresh FAVs containing essential micro and macronutrients to address their nutritional needs and reduce symptoms (6).

Few studies conducted in high-income countries among the general population, including HIV-infected adults, indicate very low to low levels of fruit and vegetable dietary intake. In the USA, only approximately 14% of the total population meets the recommended fruit intake levels and 8% for vegetables (9). Similarly, studies conducted in China and Canada report insufficient fruit and vegetable intake, with 55.2% of the labor force and 40.7% of students falling short of recommended levels, showing no significant differences between urban and rural populations in the proportion of insufficient vegetable intake (10). A study conducted in Portugal among HIV-infected adults also indicated the low frequency of fruit and vegetable consumption, in which 42.5 and 23.7% of participating individuals consumed FAVs less than once per day (11). In Africa, studies indicate that only a small proportion of people consume and meet the recommended amount. In that line, a study conducted in Kenya indicates that 51.0% of the people consumed fruits during the survey (the previous day), with a mean intake of 189.6 (16.8) g/day, of which only 16% of the participants met WHO recommendations (12). In the study conducted in Uganda among adults, it was indicated that only 12.2% of them consumed five or more servings of fruits and/or vegetables per day in a typical week (13). The evidence from a review of high-income countries indicated that the sex, age, marital status, educational status, and income of participants were found to be contributing factors to the low level of fruit and vegetable consumption (14).

A healthy diet, including FAVs, will have a significant impact not only on the quality of life of patients but also on the success of ART treatment (15). The World Health Organization (WHO) recommends a daily intake of 400 g of FAVs, equivalent to five portions, to mitigate the risk of chronic diseases. This intake is also beneficial for HIV-positive individuals, as it helps address micronutrient deficiencies, including antioxidants such as vitamins C, A, E, and selenium. These nutrients contribute to metabolic regulation and bolster immune responses, potentially slowing the progression of HIV infection (16, 17).

In this regard, in the review of existing evidence, we did not find a single study investigating fruit and vegetable dietary intake among HIV-infected adults in Africa, including Ethiopia. Furthermore, the review of national HIV and nutrition guidelines revealed that while there is a significant focus on nutritional counseling for macronutrient and micronutrient deficiency-related problems (18), there is no explicit mention of the benefits of a healthy diet, which is assumed to contribute to improvements in the immune system function and enhanced quality of life among HIV-infected individuals. Therefore, the main objective of this study is to assess the magnitude of fruit and vegetable dietary intake, estimate the amount consumed, and identify factors associated with these dietary habits among adults receiving ART in health facilities in northcentral Ethiopia.

## Materials and methods

### Study design, settings, and period

This study employed a multi-center facility cross-sectional study in health facilities located in North Shewa Zone, northcentral, Oromia, Ethiopia. The zone consists of 16 districts, including four town administrations and 12 rural districts (19). It is noteworthy that the zone comprises one referral hospital and four primary hospitals, along with 64 health centers and 275 health posts. Among these facilities, 18 health facilities (four hospitals and 14 health centers) were identified as having ART clinics to provide ART services to HIV-infected people. We conducted this study in ten health facilities, comprising four hospitals and six health centers, which were providing ART services to HIV-infected individuals with high caseloads and established ART clinics. The study was conducted from January 2021 to April 2022.

### Study population and participants

All people living with HIV (PLHIV) receiving ART in the six health centers and four hospitals, aged 18 years and older, regardless of their treatment regimen and duration of follow-up, were eligible for inclusion. However, patients with other concomitant chronic diseases, such as heart disease, hypertension, and diabetes mellitus, and those that could suppress the immune system and deteriorate nutritional status, as well as pregnant women who received ART, were excluded from this study to ensure data quality. This condition impairs their health and affects their access to sufficient FAVs. It could also exacerbate the existing suppression of the immune system caused by the presence of HIV.

### Sample size determination and sampling procedures

The sample for the current study was determined using the double-population proportion formula in EpiInfo version 7 for sample size calculation (20), focusing on the factors associated with fruit and vegetable (FAV) dietary intake as the outcome of interest. Based on a literature review, the sample size was determined by the differences in FAV dietary intake between the two populations, considering food support as a major exposure variable. Furthermore, it was noted that the proportion of HIV-infected adult patients with FAV intake without food support was 3.4%, with an adjusted odds ratio for the association between FAV intake and food support was 2.4 (21). With these figures, this study used a one-to-one (1:1) allocation ratio of unexposed to exposed, a 5% level of significance (two-sided), and a power of 80%. To account for potential non-responses, an additional 5% was added to the sample size, resulting in a total sample size of 865. Four hospitals and six health centers that provided care were included in this study after identifying all hospitals and health centers that provided ART in North Shewa, Oromia. A list of all eligible HIV-infected adults receiving ART in clinics was obtained from the patient registration book. The calculated sample was allocated proportionally to each included health facility based on the size of patient populations. Then, simple random sampling was used to select participants from the patient registration book using the SPSS (version 25) select cases menu.

### Study variables and measurement

The dependent variable for the study was FAV dietary intake in the last 30 days, assessed through the frequency of consumption using Behavioral Risk Factor Surveillance System (BRFSS) assessment tools. The frequency of consumption of two classes of fruits (whole fruits and 100% fruit juice, without sugar or other additives) and vegetables (such as green leafy vegetables, like cabbage and salads; cruciferous; marrow; starchy staples like potatoes, sweet potatoes, green peas, and others; carrots; and other vegetables) was assessed among HIV-infected adults in selected health facilities. We used 10 categories to assess the frequency of FAV consumption: never, <1 time per month, 1–3 times a month, once a week, 2–4 times a week, 5–6 times a week, once a day, 2–3 times a day, 4–5 times a day, and 6+ times a day (16, 22). The median frequency of FAV daily intake was initially calculated by converting weekly and monthly intake into daily intake. This was achieved by dividing the frequency of weekly or monthly reported intake by 7 or 30, respectively. Subsequently, the frequencies of all FAV variables were summed up to obtain the total frequency of FV intake. The median was then calculated using the total daily FAV frequency as a continuous variable (22). Then, it was dichotomous, coded as 1 if the median frequency was less than one time per day for low FAV dietary intake and 0 if the median frequency was greater than or equal to one time per day for high fruit and vegetable intake (22). In addition, the adequacy of FAV was also assessed by considering the portion size of each selected FAV. The portion sizes were then multiplied by the recommended grams to calculate the median grams based on the WHO/FAO recommendation. According to these recommendations, individuals should consume 400 or more grams of FAVs for overall health improvement, reducing the risk of certain NCDs and preventing chronic infections (17). This dietary practice contributes to metabolic regulation and bolsters immune responses, potentially slowing the progression of HIV infection due to the content of vitamins and minerals such as vitamins C, A, E, and selenium, which possess antioxidant effects (16, 17).

We considered two independent variable categories. The first category included sociodemographic and socioeconomic characteristics (age, gender, income, educational status, occupational status, ethnicity, religion, marital status, residence, and psychosocial support). The second category included nutrition, treatment, and clinical characteristics (the duration of ART treatment, WHO clinical stage, WHO treatment stage of HIV, opportunistic infections, therapeutic food support, follow-up interval, and food security status).

### Data collection tools and methods

A structured interviewer-administered questionnaire was developed to collect sociodemographic, socioeconomic, and clinical characteristics of HIV-infected adults. Additionally, we used the standard Behavioral Risk Factor Surveillance System (BRFSS) tool to assess the frequency and intake of FAV among HIV-infected adults. Patient records were extracted to collect data on some variables, such as the type of malignancy, IOs, anemia, and WHO staging. The data collection process was supervised by the principal investigator and two field supervisors. Nine health professionals (nurses and health officers) who were not working in ART clinics were recruited and trained for 2 days before being deployed for data collection. The questionnaire was pretested on 5% of the sample at Chancho Hospital to ensure its validity and reliability, which included content validation of the standard Behavioral Risk Factor Surveillance System (BRFSS) tool with local experts before adapting it for data collection. The necessary amendment was made based on the findings of the pretest before actual data collection.

### Data management and analysis

Data were exported from KoboToolbox to STATA 17 for analysis and modeling. A descriptive analysis was used to describe the characteristics of the study participants. A Poisson regression model with robust variance was fitted to identify factors associated with fruit and vegetable consumption. All factors that were associated with the outcome variable in the bivariable analysis with a *p*-value of 0.20 or less were included in the multivariable Poisson regression model. The crude and adjusted prevalence ratios, together with their corresponding 95% confidence intervals, were computed. The multicollinearity of explanatory variables was checked using the variance inflation factor, while the fitness of the model was checked using information criteria such as AIC and BIC. A *p-*value of <0.05 and the corresponding 95% confidence interval were considered statistically significant.

### Ethical consideration

Ethical clearance for the study was obtained from the Institutional Review Board of the College of Health Sciences, Addis Ababa University, under registration number 104/19/SPH. Data collection commenced only after obtaining permission from the participating hospitals and health centers. All focal personnel at the ART clinics of the respective health facilities were duly informed about the study protocols. The participants provided their written informed consent before participating in the study, after being fully briefed on its purpose. The confidentiality of the collected data was maintained by not revealing personal identifiers and locking the data in the file cabinet. Participation in this study was voluntary, with participants having the full right to opt out, or withdraw at any point from the study. This study did not cause harm to the participants except for minor discomfort during the interview process. There were no direct benefits for the participants in participating in this study. The soft copy of data entered into the computer was stored in encrypted files on password-protected computers.

## Results

### Sociodemographic and socioeconomic characteristics

A total of 858 HIV-infected adults were enrolled and completed the interview. A total of 552 (64.34%) participants belonged to the age group ≤ 40 years, with the mean age of the enrolled participants was 38.64 (±9.85 SD) years. Additionally, 527 participants (61.42%) were women, and 614 participants (71.56%) were from urban areas. A total of 778 (90.68%) participants were followers of Orthodox Christianity, and 519 participants (60.49%) were married. A total of 642 (74.82%) participants belonged to Oromo in ethnicity. A total of 198 (23.08%) HIV-infected adults were merchants, followed by housewives (191, 22.26%). A total of 379 (44.17%) HIV-infected adults had no formal education, and 291 (52.62%) participants responded that they earned a monthly income of 2,500 and above ETB and had a median monthly income of 2500ETB (IQR: 1,200, 4,730) (Table 1).

**Table 1:** Sociodemographic and economic characteristics of HIV-infected adults receiving antiretroviral therapy at health facilities in northcentral, Ethiopia, 2023.

**Variables**	**Frequency**	**Percent**
**Age of respondents**		
≤ 40 years	552	64.34
>40 years	306	35.66
**Mean (±)**	38.64 (±9.85)	
**Sex of participants**		
Male	331	38.58
Female	527	61.42
**Residence**		
Rural	244	28.44
Urban	614	71.56
**Religion of participants**		
Orthodox	778	90.68
Protestant	55	6.41
Others^*^	25	2.91
**Marital status**		
Married	519	60.49
Single	96	11.19
Divorced	111	12.94
Widowed	132	15.38
**Ethnicity of participants**		
Oromo	642	74.82
Amhara and Gurage	216	25.18
**Occupational status**		
Farmer	172	20.05
Housewife	191	22.26
Daily laborer	148	17.25
Employed	149	17.37
Merchant/others	198	23.08
**Educational status**		
No formal education	379	44.17
Primary school	239	27.86
Secondary and above	240	27.97
**Monthly income (*****n*** = **553)**		
<2,500 ETB	262	47.38
≥2,500 ETB	291	52.62
**Median monthly income (ETB)**	**Median**	**IQR**
Median (IQR)	2,500	(1,200, 4,660)

^*^Catholic, Muslim, and Wekefata.

### Socioeconomic support for HIV-infected adults

Regarding socioeconomic support, 415 (48.37%) HIV-infected adults received informal care from different caregivers; of which, 271 (65.30%) and 86 (20.72%) HIV-infected adults received economic support and psychological support, respectively. The majority, 245 (72.29%), received care either from their husbands or wives, and 438 (51.05%) HIV-infected adults had disclosed their HIV status, with 244 participants (55.71%) disclosing their HIV serostatus to their husbands or wives (Table 2).

**Table 2:** Socioeconomic support for HIV-infected adults receiving antiretroviral therapy at health facilities in northcentral, Ethiopia, 2023.

**Variables**	**Frequency**	**Percent**
**Presence caregiver (*****n*** = **858)**		
No	443	51.63
Yes	415	48.37
**Type of care received (*****n*** = **415)**		
Psychological support	86	20.72
Economic support	271	65.30
Social support and related	58	13.98
**Type of caregiver (*****n*** = **415)**		
Mother/father	55	13.25
Husband/wife	245	72.29
Children	80	19.28
Others	35	8.43
**Disclose their sero-status (*****n*** = **858)**		
No	420	48.95
Yes	438	51.05
**To whom you disclose (*****n*** = **438)**		
Mother/father	45	10.27
Husband/wife	244	55.71
Children	91	20.78
Others	58	13.24

### Clinical and food-related characteristics of HIV-infected adults

A total of 172 (20.05%) HIV-infected adults reported having eating problems during their treatment follow-up, the most common reasons being the loss of appetite (133, 77.33%), followed by oral candidiasis (33, 19.19%). Moreover, 173 participants (20.16%) developed opportunistic infections (OIs), and 43 participants (5.01%) reported receiving therapeutic feeding during the treatment follow-up. In total, 212 (24.71%) patients reported being anemic during their treatment follow-up. A total of 621 (72.38%) and 761 (88.69%) HIV-infected adults were at the WHO clinical stage one and WHO treatment stage one, respectively, while 534 (62.24%) participants reported that they received ART for more than 8 years, and the average amount of time that the studied HIV-infected adults received treatment was 9.21 years (±4.54 SD), which is considered a longer ART duration.

A total of 84 (9.76%) and 66 (7.69%) HIV-infected adults claimed to have been forced to engage in unprotected sex and had migrated from their previous place of residence to obtain daily food, respectively. A significant proportion of the HIV-infected adults reported experiencing food insecurity (288, 33.57%) (Table 3).

**Table 3:** Clinical and food-related characterisitics of HIV-infected adults receiving antiretroviral therapy at health facilities in northcentral, Ethiopia, 2023.

**Variables**	**Frequency**	**Percent**
**Presence of eating problems (*****n*** = **858)**		
No	686	79.95
Yes	172	20.05
**Causes of eating problems (*****n*** = **172)**		
Loss of appetite	133	77.33
Oral candidiasis	33	19.19
Esophageal candidiasis	6	3.49
**Presence of OIs (*****n*** = **858)**		
No	685	79.84
Yes	173	20.16
**Therapeutic food (*****n*** = **858)**		
No	815	94.99
Yes	43	5.01
**Presence of anemia (*****n*** = **858)**		
No	646	75.29
Yes	212	24.71
**Duration of HIV infection (*****n*** = **858)**		
<5 years	157	18.30
5–10 years	185	21.56
>10 years	516	60.14
**The average duration of HIV infection in years (*****n*** = **858)**	**Mean**	**SD**
Average (SD)	9.76	4.64
**WHO clinical stage (*****n*** = **858)**		
Stage one	621	72.38
Stage two and above	237	27.62
**WHO treatment stage (*****n*** = **858)**		
Stage one	761	88.69
Stage two and above	97	11.31
**Duration of ART (*****n*** = **858)**		
<4 years	138	16.08
4–8 years	186	21.68
>8 years	534	62.24
**The average duration of ART in years**	**Mean**	**SD**
Average (SD)	9.21	4.54
**Unprotected sex for daily food**		
No	764	88.73
Yes	84	9.76
**Ever migrated from home for food**		
No	792	92.31
Yes	66	7.69
**Food security status**		
Food secure	570	66.43
Food insecure	288	33.57

### Fruits and vegetable dietary intake frequency

We assessed the frequency of consumption of two classes of fruits (whole fruits and 100% fruit juice without sugar or other additives) and vegetables (green leafy and salads, cruciferous, marrow, starchy staples, carrots, and other vegetables) among HIV-infected adults. We found a very low frequency of FAV consumption among HIV-infected adults in relation to the recommended daily allowance. Only 205 (23.89%) and 177 (20.63%) of the HIV-infected adults consumed 100% fruit juice and whole fruit once per month, respectively. A total of 231 (26.92%) and 160 (18.65%) HIV-infected adults consumed green leafy vegetables and salad one time per month and 2–3 times per month, respectively. In total, 331 (38.58%) and 301 (35.08%) HIV-infected adults consumed cruciferous vegetables and starchy foods 2–3 times per month, respectively. Moreover, 289 (33.68%), 220 (25.64%), and 107 (12.47%) HIV-infected adults reported consuming marrow vegetables, carrots, and other vegetables, respectively (Table 4).

**Table 4:** FAVs intake frequency among HIV-infected adults in northcentral Ethiopia, 2023.

**Type of FAVs**	**Frequency of intake in the past 30 days (No: %) (*****n*** = **858)**									
	**Never**	**1 time per month**	**2–3 times per month**	**1–2 times per week**	**3–4 times per week**	**5–6 times per week**	**1 time per week**	**2–3 times per day**	**4–5 times per day**	**6 or more times per day**
100% fruit juice	559 (65.15)	205 (23.89)	78 (9.09)	9 (1.05)	2 (0.23)	2 (0.23)	1 (0.12)	1 (0.12)	1 (0.12)	0 (0.00)
Whole fruits	597 (69.58)	177 (20.63)	72 (8.39)	5 (0.58)	1 (0.12)	0 (0.00)	1 (0.12)	1 (0.12)	4 (0.47)	0 (0.00)
Green leafy vegetables and salad	390 (45.45)	231 (26.92)	160 (18.65)	47 (5.48)	16 (1.86)	3 (0.35)	7 (0.82)	0 (0.00)	4 (0.47)	0 (0.00)
Cruciferous vegetables	208 (24.24)	197 (22.96)	331 (38.58)	60 (6.99)	26 (2.91)	2 (0.23)	4 (0.47)	0 (0.00)	30 (3.50)	1 (0.12)
Marrow vegetables	377 (43.94)	289 (33.68)	158 (18.41)	16 (1.86)	5 (0.58)	3 (0.35)	4 (0.47)	0 (0.00)	5 (0.58)	1 (0.12)
Starchy foods	247 (28.79)	181 (21.10)	301 (35.08)	57 (6.64)	24 (2.68)	5 (0.58)	5 (0.58)	4 (0.47)	34 (3.96)	1 (0.12)
Carrots	269 (31.35)	220 (25.64)	212 (24.71)	76 (8.86)	36 (4.20)	12 (1.40)	8 (0.93)	1 (0.12)	23 (2.68)	1 (0.12)
Other vegetables	641 (74.71)	107 (12.47)	75 (8.74)	18 (2.10)	7 (0.82)	2 (0.23)	4 (0.47)	0 (0.00)	4 (0.47)	0 (0.00)
Any vegetables	221 (25.76)	326 (38.00)	192 (22.38)	32 (3.73)	32 (3.73)	5 (0.58)	9 (1.05)	1 (0.12)	39 (4.55)	1 (0.12)

### The level of fruit and vegetable dietary consumption among PLHIV

**Figure 1:**
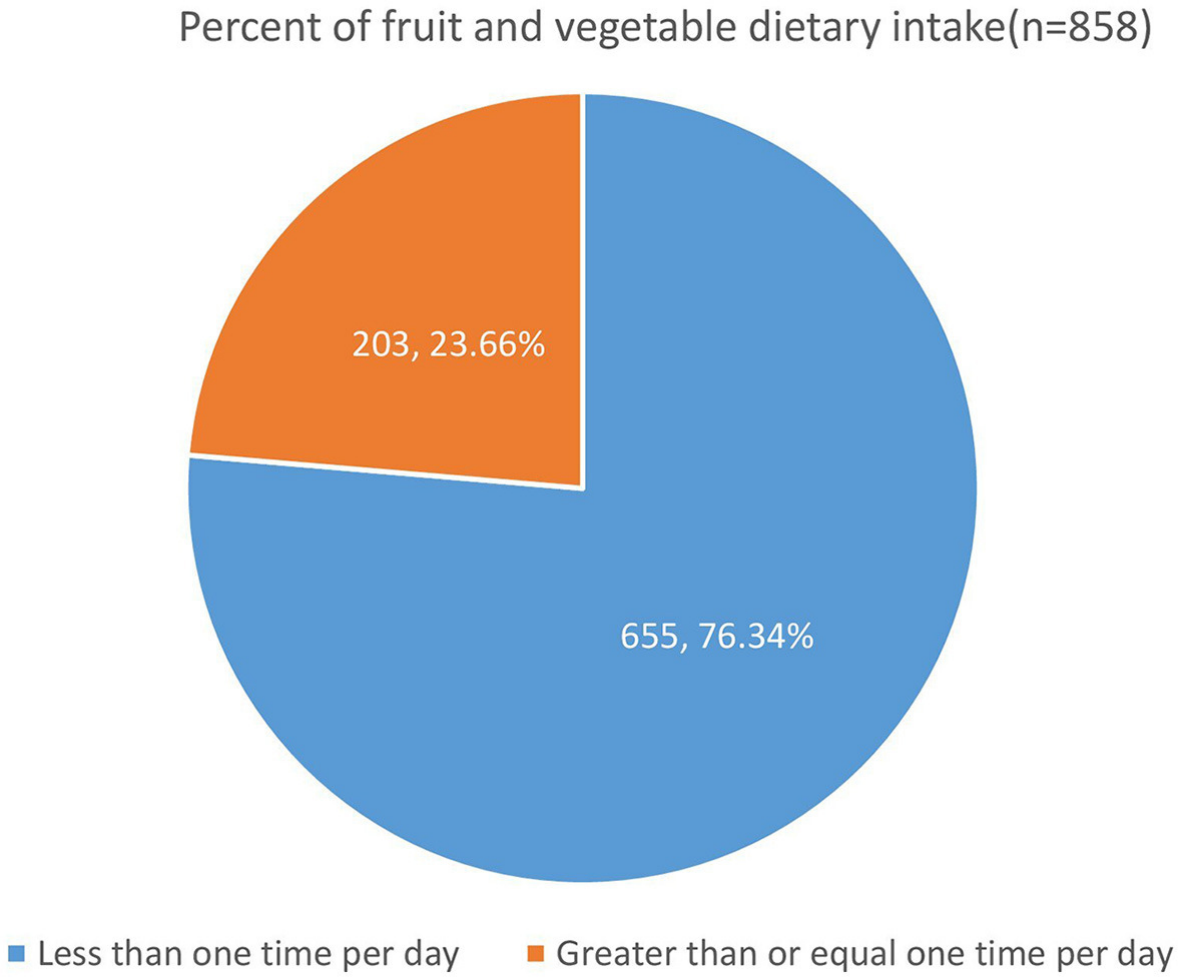
FAV dietary intake among HIV-infected adults receiving antiretroviral therapy at public health facilities in northcentral Ethiopia, 2023.

In the study, more than three-quarters, i.e., 655 [76.34%; 95% CI: (73.38, 79.07)] HIV-infected adults reported consuming FAVs less than once per day, while only 203 [23.66%; 95% CI: (20.93, 26.62)] HIV-infected adults reported consuming FAVs once per day or more (Figure 1).

**Figure 2:**
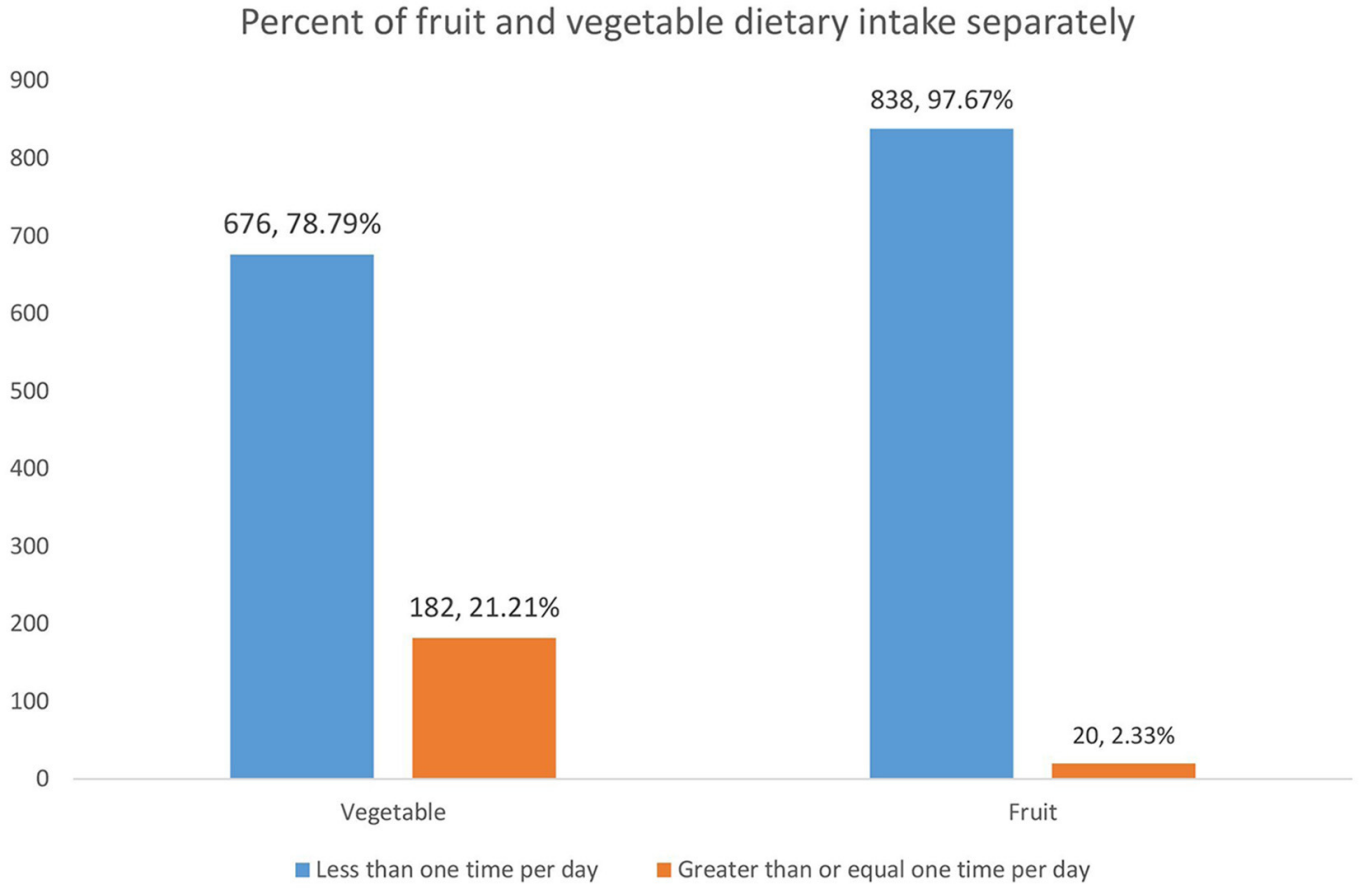
FAV dietary intake separately among HIV-infected adults receiving antiretroviral therapy at public health facilities in northcentral Ethiopia, 2023.

We also calculated the consumption of FAVs separately to compare their intake. Accordingly, 838 (97.67%, 95% CI: 96.41, 98.49) and 676 (78.79%, 95% CI: 75.92, 81.40) HIV-infected adults reported consuming fruits and vegetables less than once per day, respectively, indicating very low consumption of both fruits and vegetables (Figure 2).

### Estimated amount of fruit and vegetable consumption

In this study, we found that there was no culture of portion size estimation, including for those who reported consuming FAVs. It was very difficult to generalize since only 82 and 101 of the total sample responded to the consumption of fruits and vegetables, respectively.

Accordingly, the median (IQR) total FAV intake was 271.3 (IQR: 92.5, 439.5) g/day. The median (IQR) of the total fruit and vegetable consumption was 248.1 (IQR: 100.0, 400.0) g/day and 273.78 (IQR: 82.44, 348.33) g/day, respectively. The proportion of participants consuming 400 g or more of FAVs was calculated based only on the 108 participants whose amount of consumption was quantified. Therefore, the proportion of HIV-infected adults who consumed 400 g or more of FAV was 33 (30.6%) (Table 5).

**Table 5:** The median fruit and vegetable intake among HIV-infected adults receiving antiretroviral therapy at health facilities in North Shewa Zone, Ethiopia, 2023.

**Variables**	**Median**	**IQR**
Overall median (IQR) FAV intake (*n* = 108)	271.30 g/day	(92.5, 439.5)
Median (IQR) fruit intake (*n* = 82)	248.10 g/day	(100.0, 400.0)
Median (IQR) vegetable intake (*n* = 101)	273.78 g/day	(82.44, 348.33)

### Factors associated with fruit and vegetable dietary intake

In the bi-variable Poisson regression analysis, 14 variables, namely educational status, marital status, occupational status, monthly income, categories of caregivers, the types of care received, the presence of opportunistic infections, the presence of anemia, ever migrated from a permanent place of residence, people to whom they disclose their serostatus, duration of HIV infection, duration of ART follow-up, the WHO clinical stage, and the WHO treatment stage, showed association with a *p*-value of ≤ 0.20 and were selected as the candidates for multivariable analysis. Out of the 14 variables, the duration of HIV infection showed collinearity with other related variables but was reduced after the collinearity check using the variance inflation factor.

Consequently, the multivariable Poisson regression analysis with robust variance fitted all 13 variables simultaneously. Five of the most contributing factors were significantly and independently associated with FAV dietary intake at a 5% level of significance.

The marital status of the HIV-infected adults was significantly associated with FAV consumption, with the proportion of FAV dietary intake being 1.6 times higher among those divorced compared to those married (APR = 1.57, 95% CI: 1.16, 2.12) (Table 6).

**Table 6:** Factors associated with the magnitude of fruit and vegetable dietary intake among HIV-infected adults receiving ART at health facilities in northcentral, Ethiopia, 2023.

**Variables**	**Fruit and vegetable intake (No)**		**CPR with a 95% CI**	**APR with a 95% CI**	***P*-value**
	**High**	**Low**			
**Marital status**					
Married	123	396	1.0	1.0	
Single	36	60	0.82 (0.70, 0.96)^**^	1.28 (0.80, 2.07)	0.305
Divorced	16	95	1.12 (1.03, 1.23)^**^	**1.57 (1.16, 2.12)** ^**^	**0.003**
Widowed	28	104	1.03 (0.93, 1.14)	1.20 (0.88, 1.64)	0.239
**Occupational status**					
Farmer	42	130	1.0	1.0	
Housewife	36	155	1.07 (0.96, 1.20)^*^	1.42 (0.89, 2.27)	0.139
Daily laborer	36	112	1.00 (0.88, 1.13)	**2.08 (1.36, 3.20)** ^**^	**0.001**
Employed	41	108	0.96 (0.84, 1.09)	**1.77 (1.10 2.84)** ^**^	**0.018**
Merchant/others	48	150	1.00 (0.89, 1.13)	**1.59 (1.03, 2.47)** ^**^	**0.038**
**Type of care received**					
Psychosocial support	48	96	1.0	1.0	
Economic support	74	197	1.09 (0.95, 1.25)^*^	1.19 (0.93, 1.54)	0.172
**Care categories**					
Mother/father	23	32	1.0	1.0	
Husband/wife	**74**	**171**	1.20 (0.95, 1.52)^*^	1.14 (0.76, 1.70)	0.535
Children	12	68	1.46 (1.15, 1.86)^**^	**1.61 (1.02, 2.55)** ^**^	**0.040**
Others	13	22	1.08 (0.77, 1.52)	1.37 (0.82, 2.29)	0.234
**Ever migrated for food**					
No	181	611	1.0	1.0	
Yes	22	44	0.86 (0.73, 1.03)	0.80(0.53, 1.21)	0.291
**WHO clinical stage**					
Stage one	162	459	1.0	1.0	
Stage two & above	41	196	1.12 (1.04, 1.21)^**^	**1.32 (1.03, 1.69)** ^**^	**0.028**
**Duration of ART**					
<4 years	62	76	1.0	1.0	0.028
4–8 years	55	131	1.28 (1.07, 1.53)^**^	1.53 (0.97, 2.40)	0.066
>8 years	86	448	1.52 (1.30, 1.78)^**^	**1.78 (1.18, 2.67)** ^**^	**0.006**

Bold values indicates those factors that showed statistically significant association with FAVs dietary intake. ^*^Mean factors that show association with FAVs at *p*-value <0.25 (rule of thumb). ^**^Mean factors that show statistically significant association with FAVs at *p*-value <0.05.

The occupational status of patients was found to have a statistically significant association with FAV dietary intake. The proportion of FAV dietary intake was two times higher among daily laborers and employed patients (APR = 2.08, 95% CI: 1.36, 3.20) compared to patients who are farmers (APR = 1.77, 95% CI: 1.10, 2.84). Additionally, the proportion of FAV dietary intake was also 1.6 times higher among merchants compared to patients who are farmers (APR = 1.59, 95% CI: 1.03, 2.47) (Table 6).

The type of caregiver providing care was found to have a statistically significant association with FAV dietary intake. The proportion of FAV dietary intake was 1.6 times higher among HIV-infected adults whose caregivers are their children compared to those whose caregivers are their mothers/fathers (APR = 1.61, 95% CI: 1.02, 2.55) (Table 6).

The WHO clinical stage and the duration of antiretroviral treatment were found to be significant and independent predictors of FAV dietary intake. Specifically, HIV-infected adults at an advanced WHO clinical stage reported a 1.3 times higher FAV dietary intake compared to those at the WHO clinical stage one [APR = 1.32, 95% CI: 1.32 (1.03, 1.69)]. Similarly, the proportion of FAV dietary intake among HIV-infected adults was 1.8 times higher among those receiving ART for more than 8 years (APR = 1.78, 95% CI: 1.18, 2.67). However, the analysis did not indicate a significant association between food security status and socioeconomic support in the final Poisson multivariable regression analysis (Table 6).

## Discussion

This study aimed to determine the magnitude of FAV dietary intake, its estimated amount, and the factors associated with it among HIV-infected adults receiving ART in health facilities in northcentral Ethiopia. Accordingly, the study found that more than three-fourths [76.34%: (73.38, 79.07)] of the HIV-infected adults who participated in the study reported consuming FAV less than once per day, based on the median frequency time. This indicated very low FAV dietary intake in the studied population, particularly low fruit dietary intake. Specifically, 97.67% (96.41, 98.49) of them reported consuming fruit less than once per day, based on the median frequency. The study found a very low frequency of fruit and vegetable (FAV) consumption among HIV-infected adults in northcentral Ethiopia, which is much lower than the consumption of FAVs in the general population of Ethiopia. Approximately 15% of households reported consuming FAVs once or more per day (23, 24). The proportion of HIV-infected adults consuming FAV less than once per day in this study was higher than the findings of the study conducted in Portugal among HIV-infected individuals (42.5 and 23.7%, respectively) (16), and 63% of students of tertiary institutions from OYO State, Nigeria reported FAV intake of less than once per day (25). However, the finding is lower than that of the study conducted among adults in South Africa, where only 0.6% of adults with chronic diseases reported consuming FAV daily (26). Furthermore, the finding is higher than the findings from Five Southeast Asian Countries among the general adolescent population, where 28% reported consuming fruits less than once per day and 13.8% indicated consuming vegetables less than once per day (27).

The frequency of FAV consumption across all nine categories was low; only 23.89 and 20.63% of the HIV-infected adults consumed 100% fruit juice and whole fruit once per month, respectively. Despite the productivity of the study area, only 26.92 and 18.65% of the HIV-infected adults consumed green leafy vegetables and salad one time per month and 2–3 times per month, respectively. This finding shows that this prevalence is still lower than the prevalence found from the results of the study conducted in Portugal among a similar population, in which the highest frequency of consumption was mango, papaya, and bananas among fruits (48.75% of women and 40% of men) (16).

The study revealed that the overall median FAV intake was 271.3 g/day; of which, the median amount of fruit and vegetable intake was 248.1 and 273.8 g/day, respectively. This level of consumption was below the WHO/FAO recommendation that individuals should consume at least 400 or more grams of FAVs for their overall health improvement and chronic infection prevention (28). This finding was also lower than similar studies conducted in Kenya (12). Moreover, the results were in line with findings from studies in Thailand, in which the amounts of FAVs consumed by study participants were lower than the daily recommended amount (29, 30). The difference in FAV intake may be due to differences in the study settings and population. The WHO recommendation applies to the general population, including those with HIV, whereas our study specifically focused on HIV-infected individuals. Additionally, the study in Thailand was conducted in a middle-income country, whereas our study was conducted in a low-income country. These intake levels were also lower than those found in a study among HIV-infected pregnant women (12), suggesting that different subgroups within the HIV-infected population may have varying nutritional profiles and needs.

Marital status, occupational status, caregiver's category, the WHO clinical stage, and the duration of ART were found to be statistically significant factors for this low level of FAV dietary intake. The current study found that the marital status of HIV-infected adults was significantly associated with FAV dietary intake, with the proportion of FAV being higher among divorced people compared to those who were married. Surprisingly, we found varied evidence about the marital status of HIV-infected adults as a contributing factor for FAV dietary intake. For instance, the findings of the studies in France, Thailand, and Switzerland indicated that being single and having separate living statuses were associated with high vegetable consumption (30–32). The disparity may be due to differences in the study population and settings. The studies mentioned above were conducted in high-income countries, and the duration of the previous study was longer. Despite the variation in settings, the current finding is consistent with those from the UK, where marital transition played a significant role in fruit and vegetable consumption. Those who remained married showed significant declines in fruit quantity, fruit variety, vegetable quantity, and vegetable variety compared to those who were separated, divorced, or remained single.

The findings of this study showed that the FAV dietary intake among farmers who are producers was extremely low compared to daily laborers, employers, and merchants. This is supported by the global health and metric analysis finding, in which FAV availability has consistently been insufficient to supply recommended consumption levels (33). Additionally, similar patterns were observed in South Africa, where FAV consumption was higher among employed adults (26).

The type of caregivers who have been providing care was found to have a statistically significant association with FAV dietary intake, with caregivers who were children of HIV-infected adults and who showed higher FAV intake. This indicated that children, as caregivers for their families, will play a critical role, potentially more so than other categories of caregivers. They will also play a critical role in providing advise on various topics, including the consumption of healthy diets such as FAVs. This study's findings are consistent with those of a study conducted in peri-urban Dar es Salaam, Tanzania, where the knowledge of family members about the importance of nutritious food for HIV treatment and their support were found to play a critical role in the consumption of a healthy diet (34).

The study revealed an independent and statistically significant association between increased FAV dietary intake and advanced WHO clinical stages, as well as longer durations of antiretroviral treatment among HIV-infected adults. It was observed that the prevalence of FAV dietary intake among HIV-infected adults at an advanced WHO clinical stage and those receiving ART for more than 8 years was notably high. This correlation may be attributed to the cumulative effects of patient education and nutritional counseling provided during treatment follow-up. Patients with longer treatment follow-up durations are likely to have greater awareness of disease progression and necessary precautions due to their frequent interactions with health professionals. This study is among the very few conducted in Africa and the first to specifically explore these variables among HIV-infected adults in Ethiopia. Currently, there is a lack of comparative literature regarding the relationship between clinical factors and FAV dietary intake in this population.

We assessed the FAV dietary intake among HIV-infected adults receiving ART in health facilities in northcentral Ethiopia, using a large sample size that ensures the external validity of our findings. This pioneering study within Ethiopia among HIV-infected adults plays a crucial role in shaping nutritional counseling strategies targeted at this group. The use of the BRFSS assessment tools and the KoboToolbox digital data collection platform helps to ensure data quality. In addition, the use of a Poisson regression with a robust variance that suits prevalence ratio estimation improves the validity of the evidence generated in this particular study.

However, this study does have methodological limitations. First, since FAV consumption was assessed through participants' self-reports, there is a potential for overestimation or underestimation of actual FAV dietary intake. Second, the cross-sectional design of the study limits our ability to establish temporal relationships between outcomes and independent variables. Third, the assessment of FAV experiences of HIV-infected adults over the last 30 days is subject to recall bias, which may affect the accuracy of the reported data.

## Conclusion

The finding indicates that a very high proportion of HIV-infected adults consumed FAVs less than one time per day, which could be considered as a very low FAV dietary intake. The FAV consumption among HIV-infected adults is far below the minimum recommendation for health, which will decrease the protection against opportunistic infections and non-communicable diseases of all types. Despite their production and living in the surplus production of the area, farmers are less likely to consume FAVs. The marital status, occupational status, type of caregivers, WHO clinical stage, and duration of ART of the HIV-infected adults were found to be significant and independent contributing factors for the FAV dietary intake of HIV-infected adults.

Given these findings, there is a critical need for comprehensive, context- and culture-specific nutritional counseling to improve FAV consumption, especially among farmers. These interventions should focus on educating about portion sizes to ensure adequate daily nutrient intake. Additionally, it is essential to integrate nutritional support into the early stages of ART and throughout the treatment process. Family therapy, including counseling on healthy eating habits and the role of children as caregivers, can further support dietary improvements. We also suggest further and focused investigation of FAV dietary intake, using both quantitative and qualitative studies to address the nutritional needs of these high-risk population segments.

## Data availability statement

The raw data supporting the conclusions of this article will be made available by the authors, without undue reservation.

## Ethics statement

The study involving human participants was reviewed and approved by the study's protocol from Addis Ababa University, College of Health Sciences Institutional Review Board (IRB). Participants were informed that they could withdraw at any time and/or refrain from responding to questions. The study participants provided their written informed consent to participate in this study.

## Author contributions

DB: Conceptualization, Data curation, Formal analysis, Investigation, Methodology, Project administration, Resources, Software, Supervision, Validation, Visualization, Writing – original draft, Writing – review & editing. AA: Conceptualization, Data curation, Investigation, Methodology, Resources, Software, Supervision, Validation, Visualization, Writing – review & editing. AY: Conceptualization, Data curation, Investigation, Methodology, Resources, Software, Supervision, Validation, Visualization, Writing – review & editing. SG: Conceptualization, Data curation, Investigation, Methodology, Resources, Software, Supervision, Validation, Visualization, Writing – review & editing.
